# Past and present: a bibliometric study on polycystic ovary syndrome

**DOI:** 10.1186/s13048-022-01072-3

**Published:** 2023-02-18

**Authors:** Mengcheng Cai, Zhexin Ni, Zike Yuan, Jin Yu, Danying Zhang, Ruipin Yao, Ling Zhou, Chaoqin Yu

**Affiliations:** 1 Basic Medicine School, Naval Medical University, Shanghai, 200433 China; 2 Department of Pharmaceutical Sciences, Beijing Institute of Radiation Medicine, Beijing, 100850 China; 3 Department of Traditional Chinese Gynecology, the First Affiliated Hospital of Naval Military Medical University, Shanghai, 200433 China

**Keywords:** Polycystic ovary syndrome, Insulin resistance, Gut microbiota, Bibliometrics, Research progress, Hot spots

## Abstract

**Background:**

Polycystic ovary syndrome (PCOS) is a common gynecological endocrine disease that has a great impact on women’s physical and mental health. It is a burden to social and patients’ economy. In recent years, researchers’ understanding of PCOS has reached a new level. However, many PCOS reports have different directions, and overlapping phenomena exist. Therefore, clarifying the research status of PCOS is important. This study aims to summarise the research status of PCOS and predict the hot spots of PCOS in the future by Bibliometricx.

**Results:**

The keywords of PCOS research focused on PCOS, insulin resistance (IR), obesity and metformin. Keywords plus co-occurrence network showed that PCOS, IR and prevalence were hot spots in the recent 10 years. Moreover, we found that gut microbiota may be a carrier that can be used to study hormone levels, IR-related mechanisms, prevention and treatment in the future.

**Conclusions:**

This study is helpful for researchers to quickly grasp the current situation of PCOS research and enlighten researchers to explore new problems in PCOS.

## Background

Polycystic ovary syndrome (PCOS) is a common gynaecologic endocrine disease; its incidence rates in women were 6 to 10%, whereas those in childbearing age women were 9 to 18% [1, 2]. PCOS is characterised by hyperandrogenaemia, continuous anovulation, polycystic ovarian changes and IR [3]. Hypertrichosis, acne and obesity associated with PCOS have a great impact on women’s mental health. At the same time, irregular menstruation and amenorrhea can lead to reproductive dysfunction, which may eventually lead to infertility [4]. Currently, drug therapy for PCOS includes adjusting menstrual cycle, improving hyperandrogenaemia, IR and promoting ovulation [5]. Although drug therapy can improve the symptoms of PCOS patients, the recurrence rate is high after drug withdrawal, which causes great economic burden for patients and society. Therefore, it is very necessary to study PCOS in depth. Modern studies have shown that the aetiology of PCOS may be caused by the genes and environmental factors [6], but the specific mechanism has not been clarified.

With the deep studies on PCOS in recent years, researcher’s understanding of PCOS has reached a new level. However, a large number of PCOS reports are in different directions, and overlapping phenomena exist [7]. So, it is urgent to clarify the research status of PCOS. Although article reviews can summarise the research progress of PCOS, they lack quantitative analysis, and the number of included articles are limited. Therefore, this study aimed to grasp the research status of PCOS through bibliometrics.

Bibliometrics is used to study the status of disease and to predict the future hot spots. In the R language environment, using Bibliometricx package to analyse articles is convenient and rapid [8]. At the same time, bibliometrics is also an effective means of quickly grasping and tracking the development status of the disease, such as COVID-19, hypertension, periodontitis and others [9–11]. In this study, we used bibliometrics to analyse PCOS-related articles to provide references that researchers can use to determine the research status and predict the future hot spots of PCOS.

## Results

### Trend of annual publications and citations in PCOS research

**Fig. 1:**
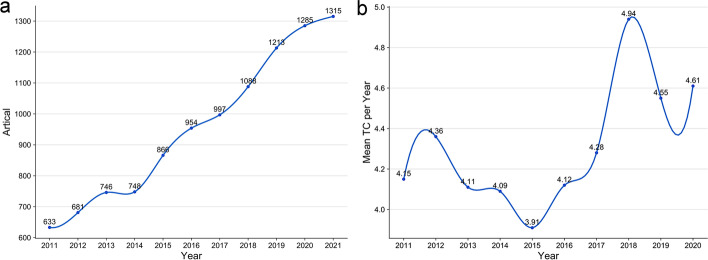
Trend of annual publications and citations in PCOS research. **a** X-axis represents the year, whereas Y-axis represents the number of PCOS-related articles published in that year. **b** X-axis represents the year, whereas Y-axis represents the average citation frequency of literature in that year

A total of 10,526 articles related to PCOS were retrieved. To grasp the annual changes in PCOS articles, we conducted a yearly statistical analysis. The PCOS articles have shown an upward trend in the past 10 years (Fig. 1a). The changes of PCOS publication from 2011 to 2021 can be divided into three stages. (1) From 2011 to 2013, the average number of articles published was 686.7, with a 9% average annual growth rate. (2) From 2013 to 2014, the average number of articles published was 747, and the number of articles presented a stable trend. (3) From 2014 to 2021, the average number of articles published was 910.75, which was significantly higher than that from 2011 to 2013, and the highest annual growth rate was 15.7%. These findings indicated that PCOS studies were gradually increasing in recent years. Research shows that the influence of articles is positively correlated with the citation frequency. Our analysis showed the trend of mean total citation per year in 2011–2020 (Fig. 1b). The average citation frequency of PCOS articles in each year was stable (about 4 to 5 times), but this can still improve.

### Journals ranking in PCOS research

**Fig. 2:**
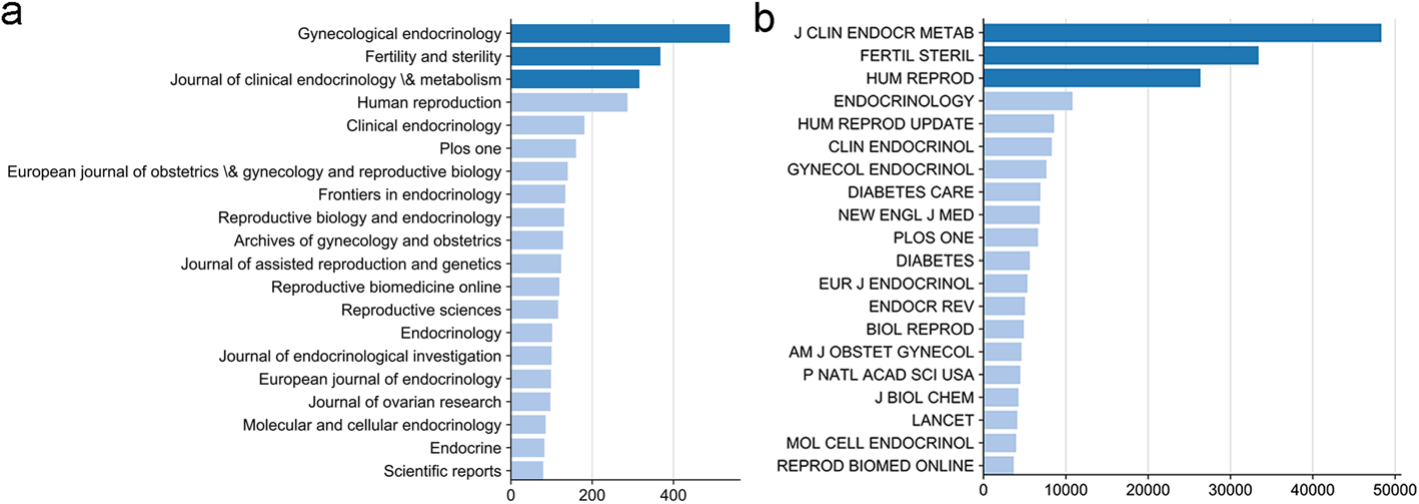
Journal ranking in PCOS research. **a** The number of articles published in the recent 10 years. X-axis represents the number of articles, whereas Y-axis represents the journal name. **b** The number of articles cited in the recent 10 years. X-axis represents the number of citations, whereas Y-axis represents the journal name

Twenty journals published the highest number of PCOS-related articles from 2011 to 2021 (Fig. 2a). The Journal of Gynecological Endocrinology had the highest number of PCOS articles with 538 articles, accounting for 5.11% (538/10,526) of the total number of articles. Secondly, Journal of Fertility and Sterility published 316 articles, accounting for 3.49% (367/10,526) of all the articles. Thirdly, Journal of Clinical Endocrinology \& Metabolism published 316 articles, accounting for 3.00% (316/10,526) of all the articles. Moreover, we analysed journal citations. The most highly cited journals include Journal of Clinical Endocrinology & Metabolism, Fertility and Sterility and Human Reproduction; these references were cited 48,287, 33,353 and 26,352 times, respectively (Fig. 2b). Furthermore, we found 22 core journals by Bradford’s law. The top 5 core journals include Gynecological Endocrinology, Fertility and Sterility, Journal of Clinical Endocrinology \& Metabolism, Human Reproduction and Clinical Endocrinology (Table 1).

**Table 1:** Core journals based on Bradford’s law

Journals	Rank	Freq
Gynecological endocrinology	1	538
Fertility and sterility	2	367
Journal of clinical endocrinology \& metabolism	3	316
Human reproduction	4	286
Clinical endocrinology	5	180
Plos one	6	160
European journal of obstetrics \& gynecology and reproductive biology	7	139
Frontiers in endocrinology	8	133
Reproductive biology and endocrinology	9	130
Archives of gynecology and obstetrics	10	127
Journal of assisted reproduction and genetics	11	123
Reproductive biomedicine online	12	118
Reproductive sciences	13	116
Endocrinology	14	101
Journal of endocrinological investigation	15	99
European journal of endocrinology	16	98
Journal of ovarian research	17	97
Molecular and cellular endocrinology	18	85
Endocrine	19	82
Scientific reports	20	79
Journal of obstetrics and gynaecology research	21	78
International journal of endocrinology	22	73

Rank represents journal position. Freq represents the number of articles published in journal

### Leader authors in PCOS research

**Fig. 3:**
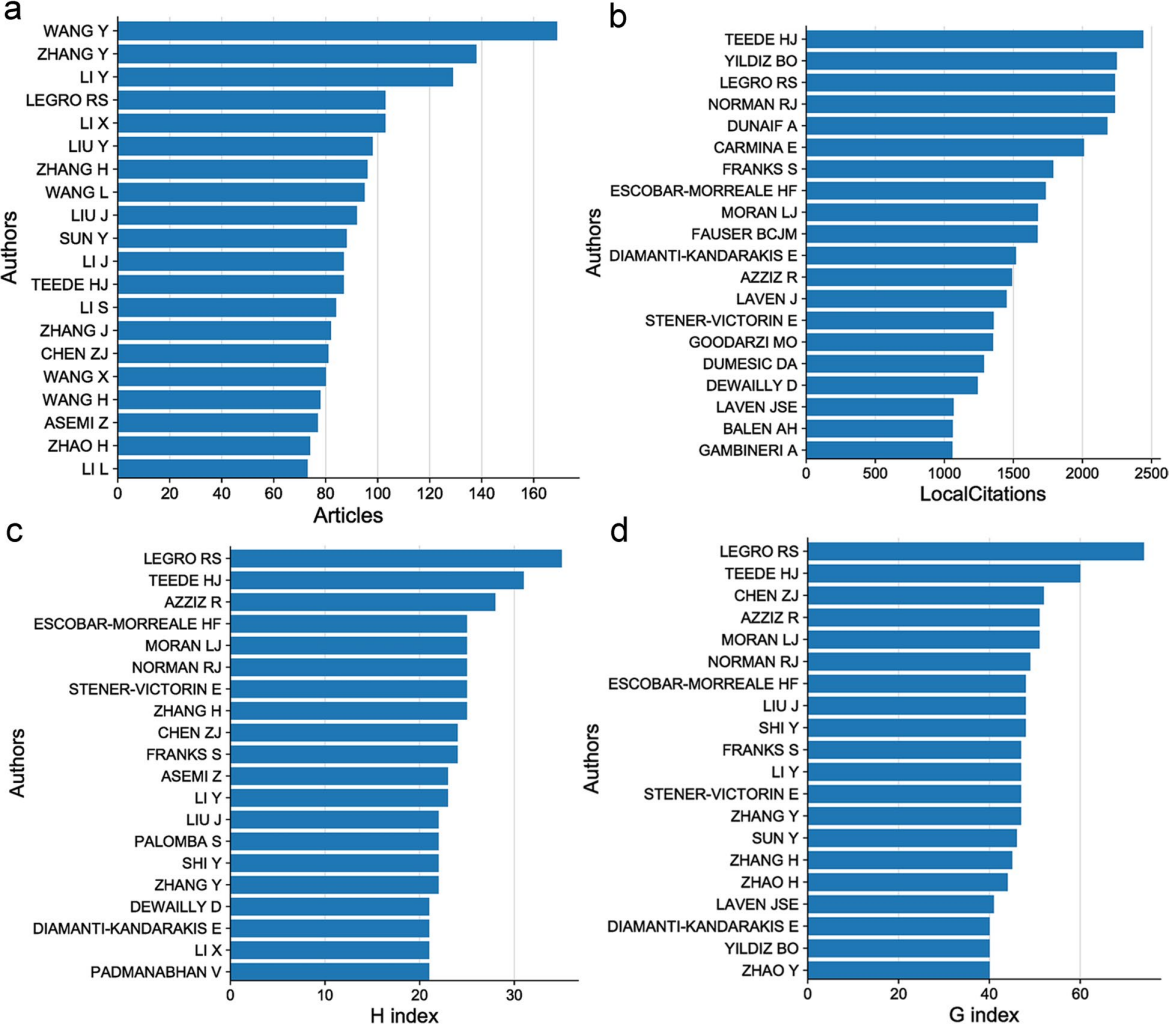
Leader authors in PCOS research. **a** The publication of the author’s articles, X-axis represents the number of articles, whereas Y-axis represents the author’s name. **b** The citation of the author’s articles. X-axis represents the cited frequency, whereas Y-axis represents the author’s name. **c** The author’s H index. X-axis represents the H index value, whereas Y-axis represents the author’s name. **d** The author’s G index. X-axis represents the G index value, whereas Y-axis represents the author’s name

From the perspective of authors, Wang Y, Zhang Y and LI Y published the most articles on PCOS (169, 138 and 129, respectively), as shown in Fig. 3a. In addition, we analysed the authors with the highest number of cited articles. TEEDEHJ, YILDIZBO and LEGRORS were cited 2441, 2249 and 2236 times, respectively (Fig. 3b). A person has N articles cited at least N times in all his academic articles, and his H index is N. Compared with the total impact factor and total citation times, H index represents the quantity and quality academic output of researchers [12]. G index is a derivative index of H index, which is proposed to compensate the H index defect. Like the H index, the larger the G index is, the greater the academic influence and academic achievement of the researcher are [13]. We analysed the author’s H and G indexes, the results showed that the top 3 authors of H index were LEGRORS, TEEDEHJ and AZZIZR, whose H indexes were 35, 31 and 28, respectively (Fig. 3c). The top 3 authors of G index were LEGRORS, TEEDEHJ and CHENZJ, whose G index were 74, 60 and 52, respectively (Fig. 3d).

### Core institutions and countries in PCOS research

**Fig. 4:**
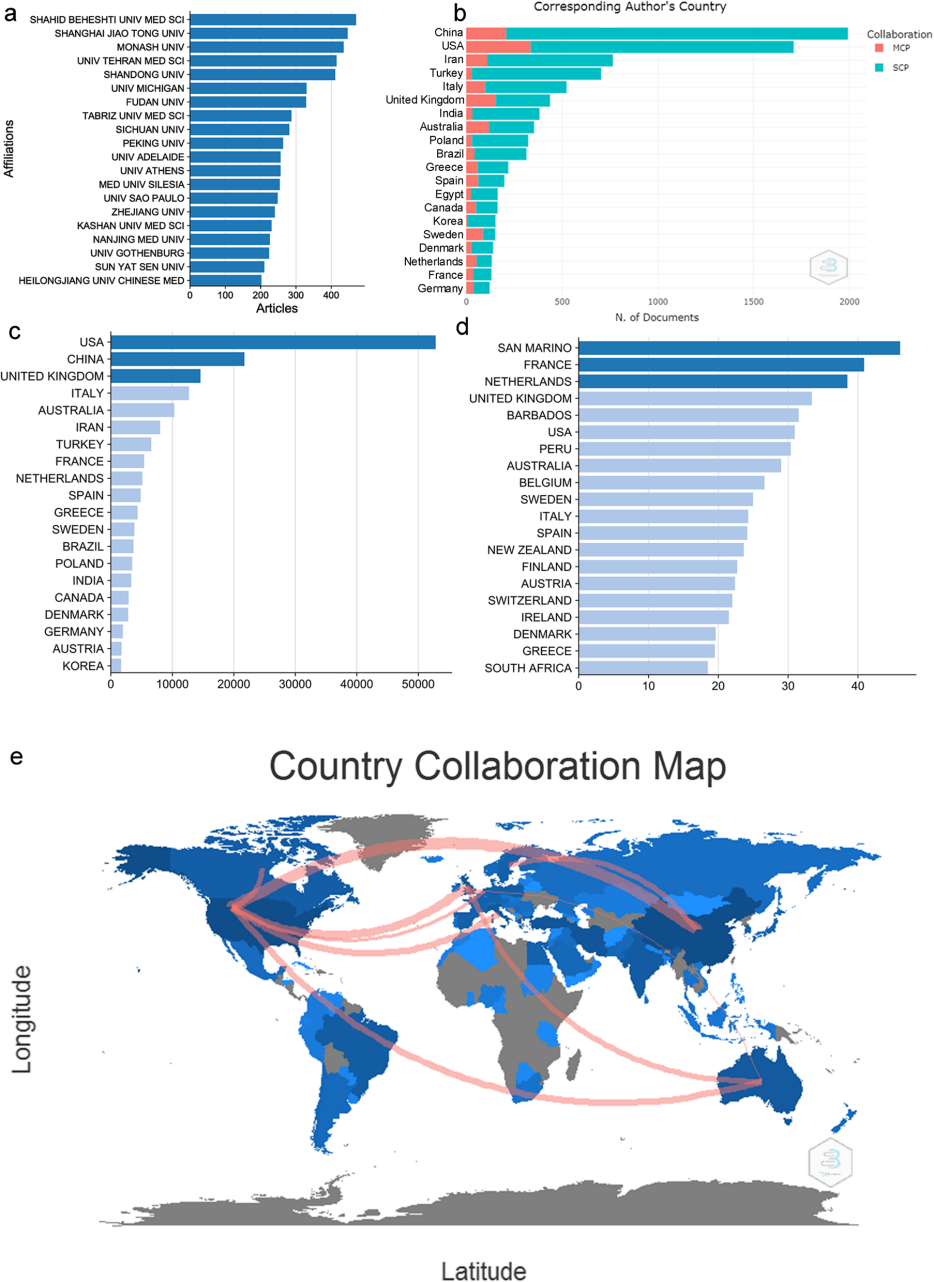
Core institutions and countries in PCOS research. **a** X-axis represents the number of articles, whereas Y-axis represents the name of the organisation. **b** The nationality of corresponding authors of PCOS articles published in various countries. X-axis represents the number of articles, whereas Y-axis represents the nationality. **c** The number of cited PCOS articles in various countries. X-axis represents the number of articles, and Y-axis represents the country. **d** The average number of cited PCOS articles of each country. X-axis represents the number of articles, and Y-axis represents the country. **e** The cooperative relationship between countries. A darker blue colour indicates that more articles were published in the region. A thicker red line represents the higher number of articles’ authors that cooperated between the two regions

From the perspective of institutions’ and countries’ publications, the top 3 publishing institutions were SHAHID BEHESHTI UNIV MED SCI, SHANG HAI JIAO TONG UNIV and MONASH UNIV (470, 446 and 435 published articles, respectively), as shown in Fig. 4a. Furthermore, we classified the nationality of corresponding authors. MCP represented the corresponding authors, and researchers had different nationalities. SCP showed that the corresponding authors and researchers came from same nationality. Results showed that there were 1992 articles come from China, ranking the first in the world; among these, 209 articles were MCP, and 1783 articles were SCP (Fig. 4b). In addition, we also analysed the countries’ article citations. The results showed that the USA had the most citation among nations (52,783 times), followed by China (21,703 times), as shown in Fig. 4c. The country with the most average number of citations per year was San Marino (46 times), followed by France (40.36 times), as shown in Fig. 4d. Furthermore, our analysis showed that China and the USA had the closest research cooperation on PCOS (Fig. 4e).

### Top 10 locally cited references in PCOS research

Locally cited references represent the references of the articles in the current data set. In our study results, Revised 2003 consensus on diagnostic criteria and long-term health risks related to polycystic ovary syndrome had the highest citation (2296 times). The next highest citation was Revised 2003 consensus on diagnostic criteria and long-term health risks related to polycystic ovary syndrome (PCOS) with 2008 times. Three of the top 10 locally cited references were from FERTIL STERIL, 3 from CLIN ENDOCR METAB and 2 from HUM REPROD (Table 2).

**Table 2:** The top 10 local cited references related to PCOS research

Document	Title	Citation
CHANG J, 2004, FERTIL STERIL [14]	Revised 2003 consensus on diagnostic criteria and long-term health risks related to polycystic ovary syndrome	2296
FAUSER BCJM, 2004, HUM REPROD [15]	Revised 2003 consensus on diagnostic criteria and long-term health risks related to polycystic ovary syndrome (PCOS)	2008
AZZIZ R, 2004, J CLIN ENDOCR METAB [16]	The prevalence and features of the polycystic ovary syndrome in an unselected population	1057
MARCH WA, 2010, HUM REPROD [17]	The prevalence of polycystic ovary syndrome in a community sample assessed under contrasting diagnostic criteria	857
AZZIZ R, 2009, FERTIL STERIL [18]	The Androgen Excess and PCOS Society criteria for the polycystic ovary syndrome: the complete task force report	841
MATTHEWS DR, 1985, DIABETOLOGIA [19]	Homeostasis model assessment: insulin resistance and beta-cell function from fasting plasma glucose and insulin concentrations in man	781
AZZIZ R, 2006, J CLIN ENDOCR METAB [20]	Positions statement: criteria for defining polycystic ovary syndrome as a predominantly hyperandrogenic syndrome: an Androgen Excess Society guideline	724
FAUSER BCJM, 2012, FERTIL STERIL [21]	Consensus on women’s health aspects of polycystic ovary syndrome (PCOS): the Amsterdam ESHRE/ASRM-Sponsored 3rd PCOS Consensus Workshop Group	673
EHRMANN DA, 2005, NEW ENGL J MED [22]	Polycystic ovary syndrome	663
LEGRO RS, 2013, J CLIN ENDOCR METAB [23]	Diagnosis and treatment of polycystic ovary syndrome: an Endocrine Society clinical practice guideline	662

### Hot spots and evolution of PCOS research

**Fig. 5:**
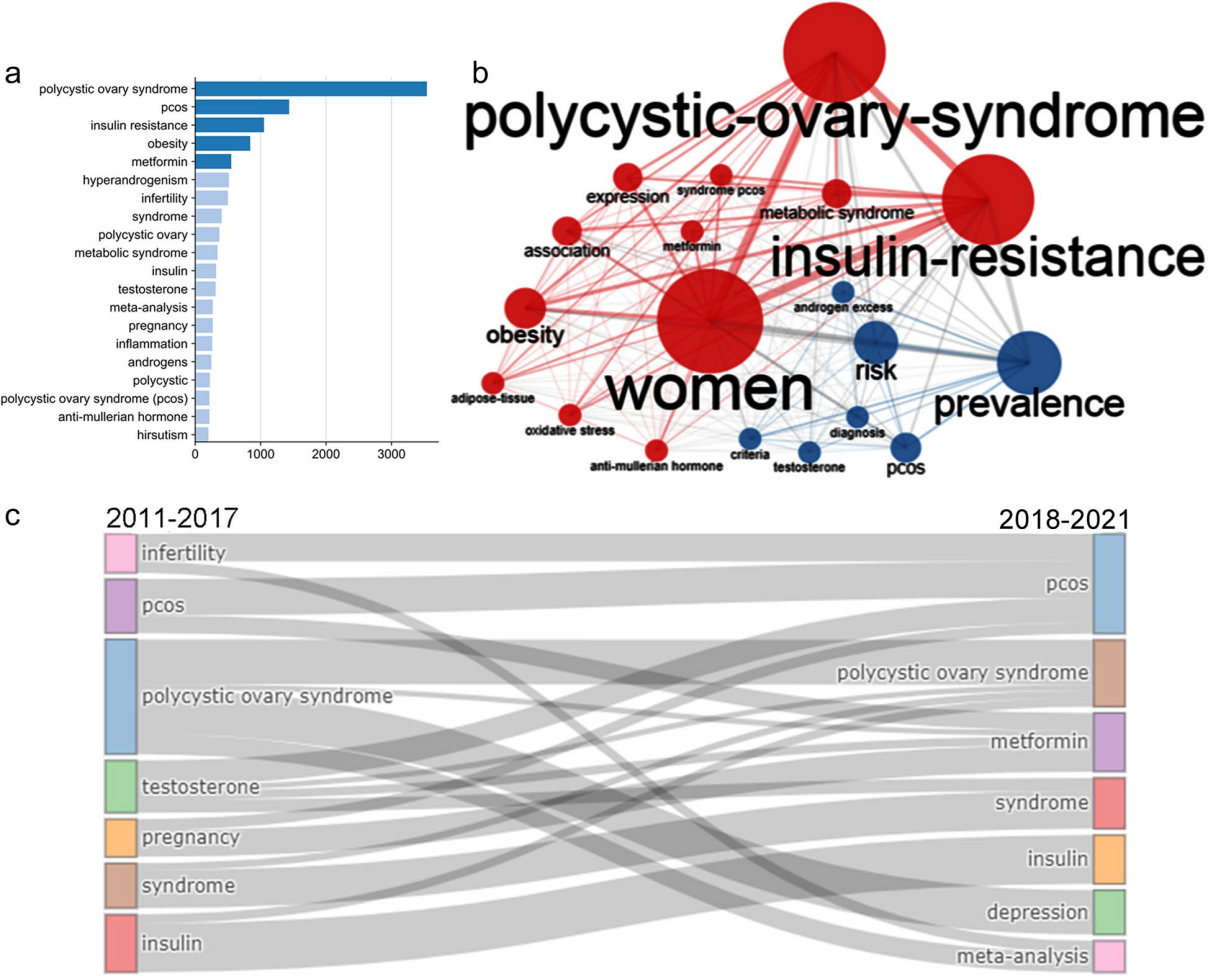
Hot spots and evolution of PCOS research. **a** Keyword word frequency. X-axis represents keyword frequency, whereas the Y-axis represents keyword. **b** Keyword plus co-occurrence network. A larger circle represents a higher frequency of keyword occurrence. A thicker line represents a higher correlation between the two keywords. **c** Keyword evolution analysis. The core keywords from 2011 to 2017 are on the left, whereas those from 2018 to 2021 are on the right

The top 5 keywords were polycystic ovary syndrome (*n* = 3538), PCOS (*n* = 1433), IR (*n* = 1045), obesity (*n* = 836) and metformin (*n* = 547). This list indicated that metabolic abnormalities in PCOS patients have gradually attracted researchers’ attention (Fig. 5a). Further analysis by keyword plus showed that the core keywords were PCOS, IR and prevalence (Fig. 5b), which again demonstrated the significance of the research on the PCOS with IR.

After clarifying the hot spots in recent years, we tried to establish the keyword evolution in different periods. Our results showed that the research on PCOS in the past 10 years focused on PCOS disease, symptoms and insulin. However, different research directions exist in different periods. The research from 2011 to 2017 focused on androgen, infertility and pregnancy. The research from 2018 to 2021 focused on metformin (therapy), depression (emotion), meta-analysis and others. The change of keywords indicated that the current research on PCOS has entered the modern medical mode (Fig. 5c).

### Prediction of PCOS future hot spots

**Fig. 6:**
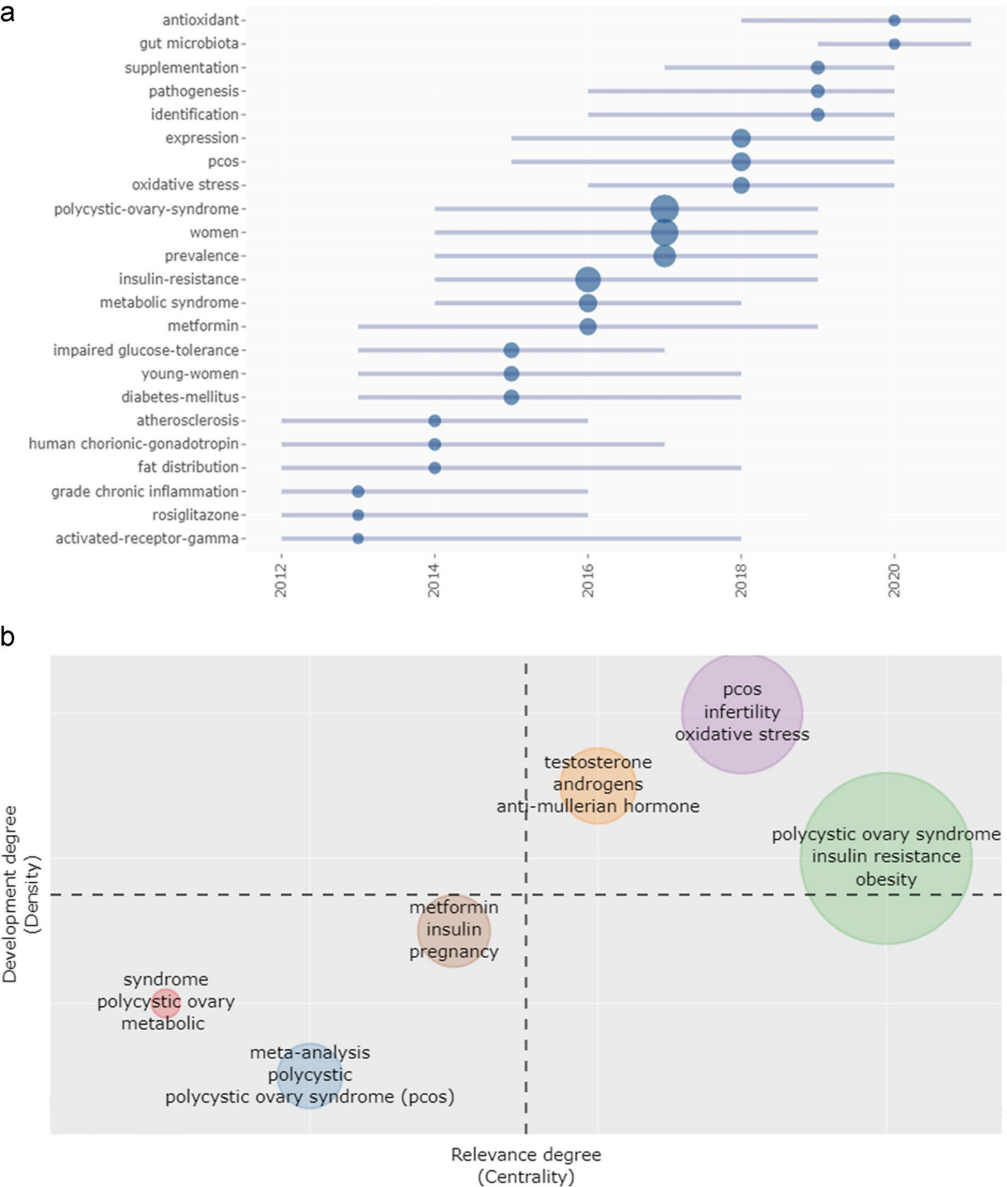
Prediction of PCOS research future hot spots. **a** Research hot spots in recent years. X-axis represents the year, whereas the Y-axis represents the hot spot. The larger the circle is, the higher the frequency of the word is. **b** Development trend and importance of hot spots

The future hot spots of PCOS may be closely related to antioxidant stress and gut microbiota (Fig. 6a). In addition, we also analysed the importance and development trends of hot spots (Fig. 6b). The first quadrant represented the important hot spots with good development problems. The second quadrant represented well-developed but unimportant problems. The third quadrant is at the edge of the theme; it represented bad development and unnecessary problems. The fourth quadrant represented the important problems that were not well developed. The hot spots were divided into three categories, as follows. Category I was hormone (including testosterone, androgen and anti-Mullerian hormone). Category II was symptom (including polycystic ovary syndrome, obesity and IR). Category III was function (including PCOS, infertility and oxidative stress). Based on the results, we speculated that the gut microbiota may be a carrier for future studies on PCOS and its mechanism.

## Discussion

In this study, Bibliometricx package was used to analysis the 10,526 PCOS related articles collected in the Web of Science database. Include analysis of the annual publications and citations trend, journal articles and citations, author, institutions, countries, locally cited reference, key words evolution and hot spots. The study hopes to provide reference for PCOS researchers.

From 2011 to 2021, the number of global PCOS articles shows an increasing trend. Based on the current growth trend, the number of PCOS-related articles was predicted to further increase in the future. Although the research on PCOS has been deepening in recent years, the number of research articles is insufficient to cover PCOS, which has such a high incidence. The average number of cited articles in PCOS was only 4–5 per year, which still represents a large gap compared with other studies [24, 25]. This number of articles shows that the influence of PCOS is insufficient at present. Thus, it is particularly important to deepen the research on PCOS. Future research on PCOS needs more input. Our results indicated that PCOS has a broad development prospect.

Our results showed that Gynecological endocrinology has the highest number of publications, in addition, Fertility and Sterility, Journal of Clinical Endocrinology & Metabolism, Human Reproduction and Clinical Endocrinology were the core journals of PCOS. In the future, frontiers and advances in PCOS may be published in these journals. Through H and G indexes, we found that Legro Richard S, Aziz Ricardo and Teede HJ are the leaders in PCOS field. Regarding the academic institution ranking, the Shahid Beheshti University Medical Center in Iran, Shanghai Jiao Tong University in China and Monash University in Australia were the academic institutions that published the highest number of articles on PCOS. Regarding country ranking, USA published the highest number of PCOS-related articles, followed by China. Britain, Italy and Australia ranked 3, 4 and 5, respectively. Therefore, these countries, institutes and researchers may continue to play an important role in future PCOS research.

Through the analysis of keywords and keyword plus, we found that IR was the focus of PCOS research in the past decade. IR is closely related to PCOS, and 50–70% of PCOS patients had IR [26]. IR can cause metabolic abnormalities and can increase the risk of type 2 diabetes and metabolic syndrome [27]. Therefore, timely and effective alleviation of IR in PCOS patients can prevent long-term complications. In addition, IR aggravates the symptoms of hyperandrogenaemia, thereby forming a vicious cycle and promoting the development of PCOS [3]. A study has shown that alleviating IR can reduce serum testosterone level, hirsutism and acne symptoms [28]. Moreover, PCOS patients with IR have significantly increased incidence of ovulation disorders, anovulation, abortion and pre-eclampsia during pregnancy [29, 30]. Alleviating IR can normalise the menstrual cycle and increase ovulation rate in PCOS patients [31]. Therefore, alleviating IR is very necessary to prevent long-term complications, improve the hormone level and restore the reproductive function of PCOS patients. These findings indicate that the results of our bibliometrics are credible.

How does PCOS IR come about? Previous studies of our group have shown that Lachnoclostridium, Fusobacterium, Coprococcus_2 and Tyzzerela 4 are the characteristic gut microbiota of obese PCOS patients [32]. *Lactococcus* is a characteristic gut microbiota of non-obese PCOS patients [33]. Drug therapy improves IR of patients, at the same time, [Eubacterium]_rectale_group, Escherichia-Shigella, Fusicatenibacter, and Megamonas intestinal bacteria also significantly change [34]. Thus, gut microbiota is closely related to PCOS IR. Furthermore, Qi X, et al. showed that gut microbiota of PCOS patients significantly increased *Bacteroides vulgatus*, and transplantation of *B. vulgatus* into mice resulted in ovarian dysfunction, IR and infertility [35]. These results confirmed that gut microbiota disorder can lead to PCOS IR. Giampaolino P, et al. searched PubMed and Medline databases and found that PCOS IR was related to gut microbiota [36]. PCOS IR is due to the imbalance of gut microbiota, and further study of gut microbiota may lead to the discovery of a new mechanism of PCOS IR.

Gut microbiota is at the core of multi-angle research in PCOS. Oxidative stress, metabolomics and inflammatory response of PCOS can all be studied through gut microbiota research. Li T showed that a superoxide dismutase mimic can regulate gut microbiota and reduce intestinal oxidative stress to treat PCOS [37]. He Y, et al. showed that *Staphylococcus* and *Lactobacillus* are associated with sex hormone levels. Through the treatment of gut microbiota, some sex hormones were restored [38]. In recent years, more and more studies have shown that PCOS is a chronic low-grade inflammatory disease [39, 40] closely related to gut microbiota. Studies have shown that dysregulation of gut microbiota reduces the function of intestinal tight junctions, leading to LPS translocation [41, 42]. LPS may function as an early factor of inflammation and IR by activating TLR4 [43]. Xue J, et al. showed that inulin and metformin can regulate the gut microbiota to relieve the chronic low-grade inflammatory state of PCOS [44].

In addition to the surface research of PCOS, the mechanism has also been discussed in recent years: A research innovatively proposed that intestinal bacteria–bile acid–IL22 axis is a new mechanism to regulate PCOS [35]. James M Baker studied the oestrogen–gut microbiota axis [45]. Wang T, et al. studied the effect of steroid hormone–intestinal microbiota–inflammatory axis on PCOS in rats [46]. Liang Z et al. discussed PCOS patients based on the gut brain axis [47]. However, the mechanism of PCOS needs to be further clarified. Gut microbiota can be used as a carrier for various research directions. In our hot spot prediction, we found that gut microbiota may be a carrier for studying hormone levels, IR-related mechanisms, prevention and treatment in the future.

In this study, R language was used to conduct bibliometric analysis on PCOS articles, which can provide references for researchers to monitor current research progress and future research hot spots. Some deficiencies exist in our research. Firstly, we only selected Web of Science as the article retrieval source, and we did not search PubMed and Medline databases, which could have led to the loss of some articles. However, our study is still significant for the summary of PCOS research, because more than 10,000 articles were included. Secondly, the authors were not subdivided in our study, which can result in some authors having the same name. One author’s name may correspond to a different author. This is especially significant for Chinese authors and could result in the discrepancy of the data of the authors’ publications. However, the H and G indexes are the gold standard. Thirdly, this study was retrieved at a certain period, and the database was updated. Thus, the amount of data may be inconsistent. Fourthly, although we have made hot spot predictions for future research, further analysis is needed to confirm whether our prediction is accurate.

## Conclusions

In summary, bibliometrics research results showed that the number of PCOS articles in the world from 2010 to 2021 increased yearly. However, the average citation frequency of articles was not high. Gynecological Endocrinology had the most publications, and Journal of Clinical Endocrinology & Metabolism had the most citations. Revised 2003 consensus on diagnostic criteria and long-term health risks related to polycystic ovary syndrome was the most cited article in local references. The Shahid Beheshti University Medical Center is the most prolific institution, China is the country with the largest number of articles, and the USA is the country with the most citations. Legro Richard S, Azziz Ricardo and Teede HJ are research leaders in the PCOS field. In the past decade, studies on PCOS have focused on PCOS IR. Gut microbiota may be a carrier that can be used to study hormone levels, IR related mechanisms, prevention and treatment in the future.

## Methods

### Data retrieval

We used “Polycystic Ovary Syndrome” as the main topic in the Web of Science (https://www.webofscience.com/wos/alldb/basic-search). Web of Science Core Collection was selected for the database. The retrieval time limit was 2011–2021. The language was English. The literature type was articles and reviews [48]. In this analysis, we searched and analysed data retrospectively. So, no ethical issues were present.

### Data export

The retrieved articles were exported in bib format. One file comprised 500 articles, and then, the exported files were merged. The merged files included authors, title, document source, document type, authors’ keywords, keywords plus (assigned by the Web of Science machine learning algorithm), abstract, authors’ affiliations, corresponding author’s affiliation, cited References, total citations, publication year, DOI and subject category [8]. This step was independently exported by the two authors and checked one by one after all files were exported.

### Data analysis

**Fig. 7:**
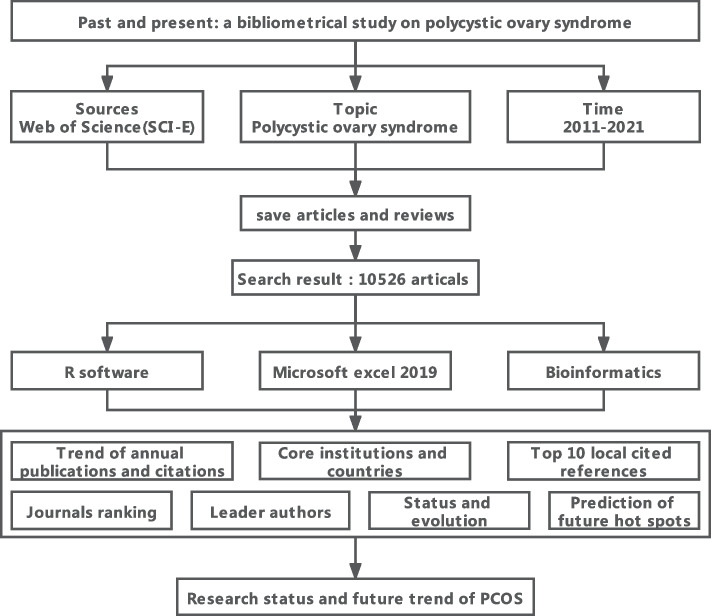
Work-flow of PCOS hot spots and future trend analysis based on Bibliometrics

Furthermore, the Bibliometricx package in R software was used to analyse the articles [49] and included the following: (1) annual publication and citation trend analysis to clarify the status of PCOS; (2) journal publications and citation analysis to clarify the core journals of PCOS; (3) authors’ publications, citations, H index and G index analysis to identify the leaders in PCOS field; (4) institution and country analyses to clarify the cooperation relationship between each institution and country in PCOS research; (5) analysis of local citation reference to obtain articles that play a leading role in the PCOS; and (6) keywords and keywords plus analysis to clarify the current development status of PCOS and predict the hot spots of PCOS research in the future. The data were optimised, and images were created using bioinformatics (http://www.bioinformatics.com.cn/). The workflow showed the PCOS research hot spots and future trend analysis based on bibliometrics (Fig. 7).

## Data Availability

The datasets used and/or analyzed during the current study are available from the corresponding author on reasonable request.

## Abbreviations

PCOS: Polycystic ovary syndrome
IR: Insulin resistance
